# An Old Story Retold: Loss of G1 Control Defines A Distinct Genomic Subtype of Esophageal Squamous Cell Carcinoma

**DOI:** 10.1016/j.gpb.2015.06.003

**Published:** 2015-09-16

**Authors:** Qiyan Wang, Jian Bai, Amir Abliz, Ying Liu, Kenan Gong, Jingjing Li, Wenjie Shi, Yaqi Pan, Fangfang Liu, Shujuan Lai, Haijun Yang, Changdong Lu, Lixin Zhang, Wei Chen, Ruiping Xu, Hong Cai, Yang Ke, Changqing Zeng

**Affiliations:** 1 MOE Key Laboratory of Carcinogenesis and Translational Research, Laboratory of Genetics, Peking University, Cancer Hospital & Institute, Beijing 100142, China; 2 CAS Key Laboratory of Genomic and Precision Medicine, Beijing Institute of Genomics, Chinese Academy of Sciences, Beijing 100101, China; 3 University of Chinese Academy of Sciences, Beijing 100049, China; 4 Anyang Cancer Hospital, Anyang 455000, China; 5 Beijing University of Chinese Medicine, Beijing 100029, China

**Keywords:** Esophageal squamous cell carcinoma, Genomic subtype, Somatic mutation, Copy number alteration, Cell cycle deregulation

## Abstract

**Esophageal squamous cell carcinoma** (ESCC) has a high mortality rate. To determine the molecular basis of ESCC development, this study sought to identify characteristic genome-wide alterations in ESCC, including exonic mutations and structural alterations. The clinical implications of these genetic alterations were also analyzed. Exome sequencing and verification were performed for nine pairs of ESCC and the matched blood samples, followed by validation with additional samples using Sanger sequencing. Whole-genome SNP arrays were employed to detect **copy number alteration** (CNA) and loss of heterozygosity (LOH) in 55 cases, including the nine ESCC samples subjected to exome sequencing. A total of 108 non-synonymous **somatic mutations** (NSSMs) in 102 genes were verified in nine patients. The chromatin modification process was found to be enriched in our gene ontology (GO) analysis. Tumor genomes with *TP53* mutations were significantly more unstable than those without *TP53* mutations. In terms of the landscape of genomic alterations, deletion of 9p21.3 covering *CDKN2A/2B* (30.9%), amplification of 11q13.3 covering *CCND1* (30.9%), and *TP53* point mutation (50.9%) occurred in two-thirds of the cases. These results suggest that the deregulation of the G1 phase during the cell cycle is a key event in ESCC. Furthermore, six minimal common regions were found to be significantly altered in ESCC samples and three of them, 9p21.3, 7p11.2, and 3p12.1, were associated with lymph node metastasis. With the high correlation of *TP53* mutation and genomic instability in ESCC, the amplification of *CCND1*, the deletion of *CDKN2A/2B*, and the **somatic mutation** of *TP53* appear to play pivotal roles via G1 deregulation and therefore helps to classify this cancer into different **genomic subtypes**. These findings provide clinical significance that could be useful in future molecular diagnoses and therapeutic targeting.

## Introduction

Esophageal cancer is a common type of cancer that is strongly associated with high mortality and ranks as the sixth leading cause of death from cancer [1]. Squamous cell carcinoma and adenocarcinoma are the major types of esophageal cancer. In the area with high prevalence of esophageal cancer (also known as the “esophageal cancer belt”), which stretches from Northern Iran through the Central Asia to the North Central China, 90% of all cases are diagnosed as squamous cell carcinomas [2–4]. The 5-year survival rate for esophageal squamous cell carcinoma (ESCC) has been low, but the principal causes for this disease remain elusive [5–7]. Previous studies have demonstrated a series of genetic alterations, including a high somatic mutation rate for *TP53* and genomic instability in numerous chromosomes [8–13]. A comprehensive description of various types of genetic alterations in ESCC and their correlation with clinical outcome would be a great step forward in our understanding of the mechanism involved in ESCC development, and could be applied to improve the survival rate of patients.

In this study, we analyzed the ESCC genome by conducting exome sequencing of nine ESCC sample pairs along with whole-genome SNP arrays of 55 tumor samples in total. Our results revealed a very high correlation of *TP53* somatic mutation with genomic instabilities in ESCC. Interruption of G1 control by *TP53* somatic mutation and copy number alterations (CNAs) was found in over 65% of ESCC cases. Furthermore, for the first time we have identified a significant correlation between copy number aberrations in three minimal common regions (MCRs), *i.e.*, amplification of 7p11.2, deletion of 3p12.1, and deletion of 9p21.3, and lymph node metastases in ESCC (*P* < 0.05).

## Results

### High heterogeneity of the somatic mutation spectrum in ESCC

**Supplementary Fig. S1:**
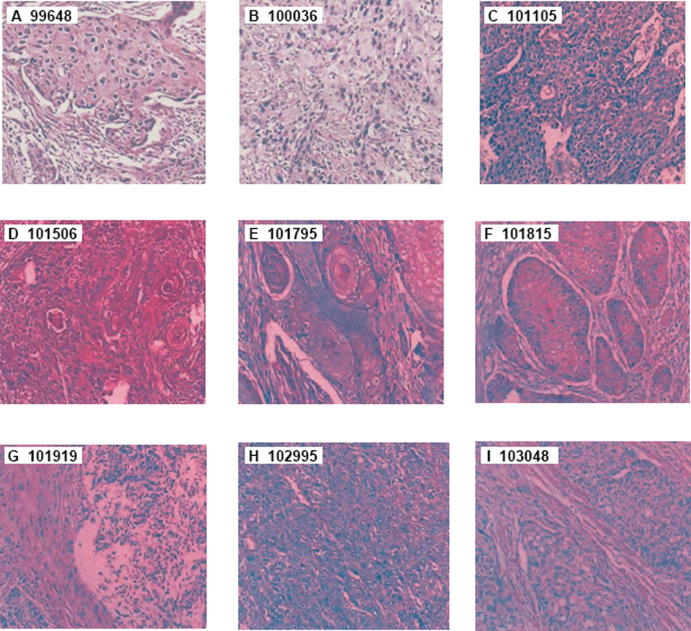
**Hematoxylin and eosin staining of nine tumor samples subjected to exome sequencing**. The nine ESCC samples subjected to exome sequencing were examined using hematoxylin and eosin staining. Tumor cell content of each sample (as indicated by patient ID) was evaluated and reviewed by two pathologists independently. The magnification for all of the images was 400×.

**Supplementary Fig. S2:**
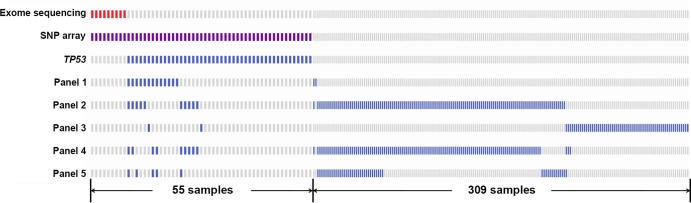
**The illustration of 364 samples used in this study**. Each small vertical bar in the same column represents an individual case. The bar width of the first 55 samples subjected to SNP assay is twice of that of the remaining 309 samples. Red bars denote the nine samples subjected to exome sequencing, and purple bars denote the 55 samples subjected to whole-genome SNP array. Samples tested by Sanger sequencing for the verification of somatic mutation in *TP53* were represented in blue bars. Sanger sequencing panels are defined as: Panel 1: *CHD3, MLL, NASP, PHF16, UBN2*; Panel 2: *CD40LG, FBXL4, INHBC, FBXW7, ANG*; Panel 3: *WNT2B, TSPYL2, SMARCD3*; Panel 4: *BRAF, AKT1, PIK3CA*; and Panel 5: *XRCC2*. Colored bars indicate the samples that were enrolled in the specific sequencing or array panels, while grey bars indicate the samples that were not included. Samples enrolled in each panel were picked randomly.

To identify the mutation profile in ESCC, exome sequencing was conducted for tumor and the matched blood samples from nine ESCC patients (further information about the patients enrolled in this study is described in Materials and methods, Figures S1 and S2, and Table S1). As summarized in Table S2, after quality control, we obtained 31 × coverage on average for the targeted bases and over 93% of all bases were covered in the targeted regions. The non-synonymous somatic mutations (NSSMs, present in tumor but absent in blood samples) identified by exome sequencing were validated using either Sequenom MassARRAY or Sanger sequencing in both tumor and blood samples. Overall, 108 NSSMs in 102 genes were verified (Table S3). The number of somatic mutations per tumor sample was highly variable (range 0–36 per sample, no somatic mutation identified in 3 samples). The most common type of mutations was missense (80, 74.07%), as compared with other types of mutations such as nonsense mutations (12, 11.11%) and small indels (14, 12.96%). Only one single splice-site (0.93%) and one read-through mutations (0.93%) were detected, respectively.

**Supplementary Fig. S3:**
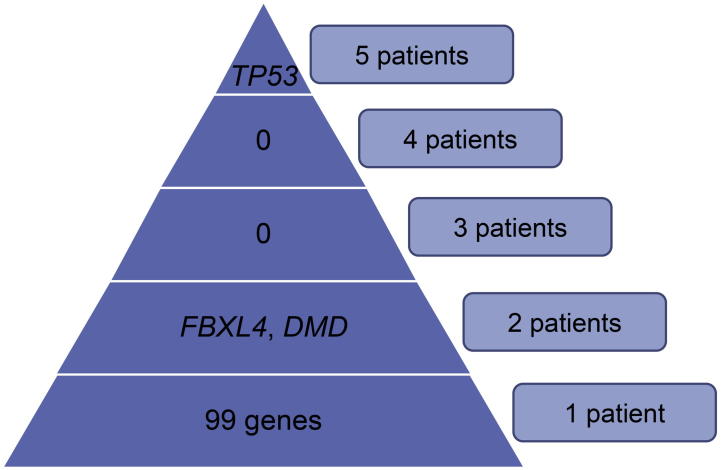
**Genes with somatic point mutations in ESCC patients identified by exome sequencing**. Only *TP53* mutations were seen in five samples, while *FBXL4* and *DMD* had recurrent mutations recovered in 2 samples.

To screen for recurrently-mutated genes, we looked into the genes with mutations in two or more of the nine tumor samples (Figure S3). As a result, somatic mutations in *TP53* were identified in five tumor samples (5/9, 55.6%). Both *FBXL4* and *DMD* were mutated in two tumor samples. *DMD* was excluded from our further analysis since this is the largest gene (measuring 2.4 Mb in human genome according to RefSeq summary). In general, random mutations may occur more frequently in larger genes, as supported by our observation of numerous mutations detected in *DMD* in non-cancerous tissues and in other datasets. *TP53* and *FBXL4* were subjected to Sanger sequencing in additional 46 and 120 samples, respectively (TP53 and Panel 2, Figure S2). 50% of the validated samples (23/46) were found to carry at least one NSSM in *TP53*. Totally 26 somatic mutations were identified in these 23 patients, among which 3 patients carried two different mutations in *TP53*. These included 19 missense mutations, 5 truncations, and 2 single nucleotide insertions that possibly result in frameshift. The possible consequences of the 19 missense mutations were predicted using PolyPhen 2 [14] and are listed in Table S4. Notably, at least one *TP53* mutation in each of the 23 patients was predicted to be deleterious. Only one of the 26 somatic mutations in *TP53* was reported in dbSNP, a database of single nucleotide polymorphisms, with a very low frequency (rs201382018, identified in patient 109596, the allele frequency is 0.02% or 1/5008, accordingly). With inclusion of the discovery set, somatic mutations in *TP53* were observed in 28/55 of ESCCs (50.90%, Tables S3 and S4). Despite the fact that there was no particular hot spot identified, most somatic mutations were localized in exons 4–8 of *TP53*. On the other hand, only two somatic missense mutations were found in *FBXL4* in the additional 120 samples of ESCCs, resulting in a total mutation rate of 3.1% in all the samples examined.

Furthermore, we conducted Sanger sequencing on the coding regions of several genes for the validation samples. These genes were selected based on their known cellular functions or their roles in various cancers. First, we examined *FBXW7*, *CD40LG*, *ANG*, and *INHBC* (Panel 2), *WNT2B* (Panel 3), and *XRCC2* (Panel 5) as shown in Figure S2, because mutations in these genes were detected in the discovery sample set (one out of nine patients, Table S3). However, no mutation was seen in the additional 120 samples. Next, we examined mutational regions or “hot spots,” including exon 4 of *AKT1*, exons 15 and 19 of *BRAF*, and exons 9 and 20 of *PIK3CA* in 120 cases (Panel 4, Figure S2). We failed to identify any somatic mutations in the regions examined either.

Taken together, the somatic mutation spectrum showed very high heterogeneity in ESCC between different tumor samples. With the exception of *TP53*, some known cancer-related genes, including *FBXW7*, *AKT1*, *BRAF*, and *PIK3CA*, showed very low mutation rates in our sample population (up to 120 samples).

### Mutations involved in chromatin modification process

To explore the biological processes that were interrupted in ESCC patients, we performed a GO cluster analysis on all the somatically-mutated genes detected in this study. Ten genes involved in chromosome organization were significantly clustered (*P* = 0.002, Table S5). In particular, seven of them (identified in five patients), including *CHD3*, *MLL*, *NASP*, *PHF16*, *SMARCD3*, *TSPYL2*, and *UBN1*, were further enriched in the chromatin modification subset (*P* = 0.005). Among these seven missense mutations, six mutations were predicted by SIFT and PolyPhen 2 to have a deleterious effect on the protein function (Table S3), suggesting that there is a frequent disruption of the chromatin modification processes in the pathogenesis of ESCC.

Considering that mutations of chromatin remodeling genes have been highlighted in several cancer studies [15–19], we then conducted Sanger sequencing for all the coding regions of these seven genes in different validation sets (samples for validation were selected randomly, however, due to limited volume of DNA samples of each patient, we cannot test all genes in a single validation set). *CHD3, MLL, NASP, PHF16,* and *UBN1* were tested in 16 samples (Panel 1), whereas *TSPYL2* and *SMARCD3* were tested in 120 samples (Panel 3) as indicated in Figure S2. However, no additional mutations in any of the validation samples were detected. This is most likely due to insufficient number of genes tested, in view of the fact that there are over 270 genes involved in chromatin modification, according to GO annotation.

### A complex landscape of structural alterations

**Supplementary Fig. S4:**
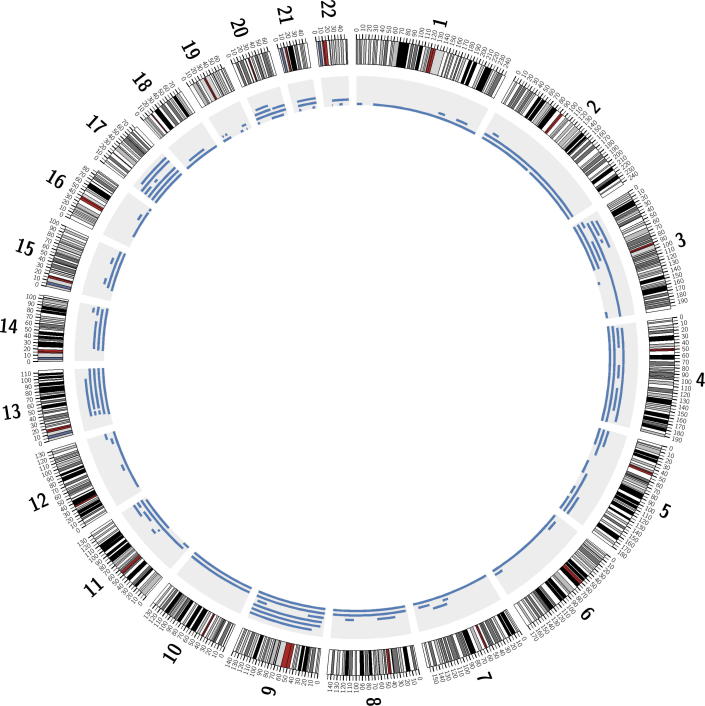
**Circular plot showing 107 imbalanced allelic events in autosomes identified by exome sequencing**. The outer ring shows autosomes 1–22 with the respective centromeres marked in red. Events and positions of allelic imbalance on each autosome are indicated by the blue bars in the grey inner ring. Blue bars were stacked up when alteration events occurred at overlapping genomic positions.

**Figure 1:**
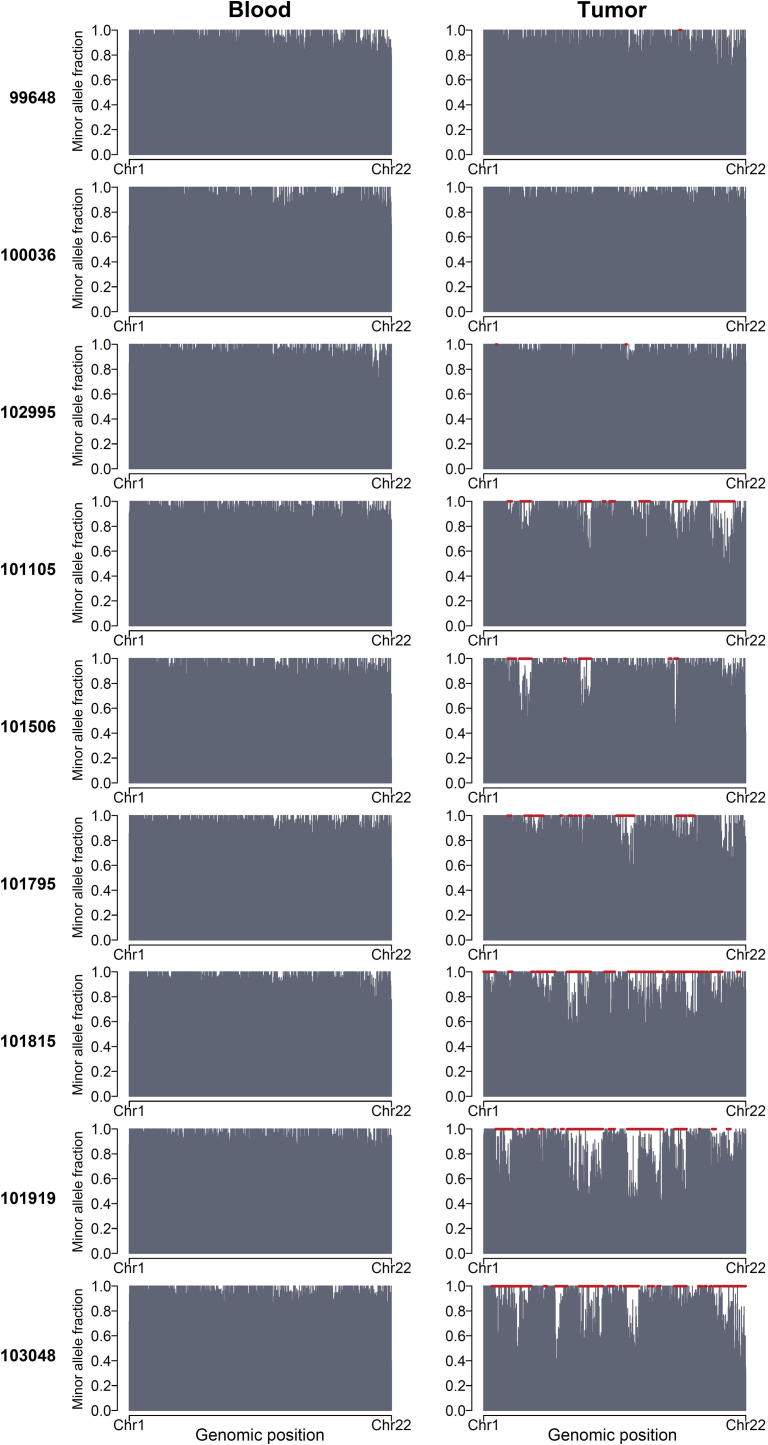
Allelic imbalance in nine ESCC sample pairs detected using exome sequencing Bar plots illustrating the minor allele fraction (MAF) of informative SNPs in each exome of blood and tumor samples. The *X*-axis indicates the genomic position of each informative SNP on autosomes. The imbalanced regions revealed by window-sliding method are indicated in red. Apparently tumor samples from patients 99648, 100036, and 102995 were rarely affected by any alteration characterized by allelic imbalance as seen in the other six tumor samples.

We used allelic imbalance as an indicator of chromosomal alterations in ESCC. A total of 107 regions of allelic imbalance were identified using window sliding, with estimated sizes in the range of 2–241 Mb (Figure S4). Two major patterns were found in these nine ESCCs, as illustrated in Figure 1. Tumor samples from patients 99648, 100036, and 102995 appeared to suffer few or no chromosomal alterations. On the other hand, the remaining 6 tumor samples showed many regions of allelic imbalance, including a few large regions. For instance, in the tumor sample from patient 101919, 22 allelic imbalance regions were identified, and the region located at Chromosome 8 measured ∼144 Mb, almost covering the whole chromosome.

Considering the difficulty in determining the boundary and copy number status in exome sequencing data, we next performed SNP arrays on 55 tumors and 9 blood samples, including the 9 tumors and 7 blood samples that were evaluated by exome sequencing, in order to further characterize the genomic aberrations in ESCC. The fraction of copy number gain, copy number loss, and copy number neutral loss of heterozygosity (CNNLOH) were then calculated in order to estimate the genome instability in each sample.

**Supplementary Fig. S5:**
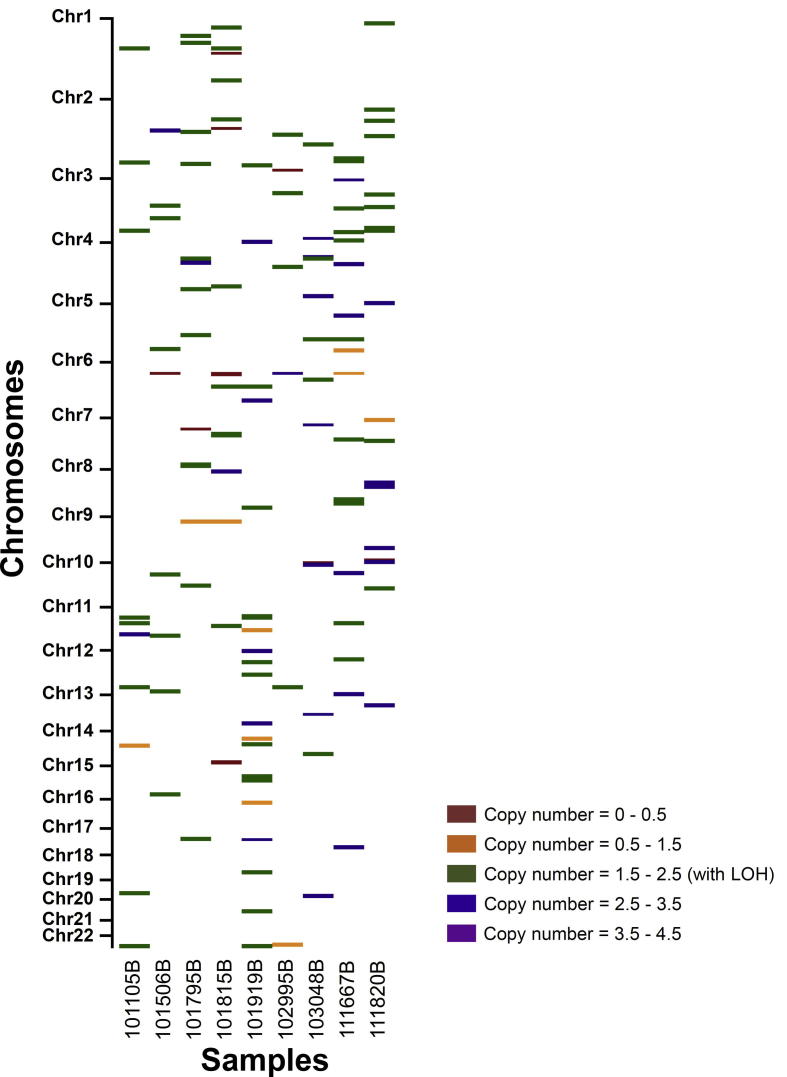
**Low genomic alterations in nine blood samples from ESCC patients identified by using cnvPartition**. Across autosomes 1−22, regions with CNAs are indicated with colored bars as follows: regions with copy number = 0–0.5 (red), 0.5–1.5 (orange), 1.5–2.5 (CNNLOH, green), 2.5–3.5 (blue), and 3.5–4.5 (purple). Patient ID was listed below each sample. CNA, copy number alteration.

As shown in Table S6, the fraction of genomic instability, including copy number gain, copy number loss, and CNNLOH, ranged from 0.001 to 0.97 (median, 0.557) among the 55 tumor samples. In total, eight tumor samples showed a genomic fraction of structural variation of less than 0.05, including the three tumor samples that showed very low allelic imbalance in exome sequencing as shown in Figure 1. Moreover, 30 of the 55 tumors (54.5%) had genome-wide alteration fractions higher than 0.5 (Table S6), suggesting severe genomic instability in those ESCC samples. In contrast to the tumors, the median fraction of altered regions in the blood samples tested was 1.8 × 10^−4^ (in the range of 0–0.06, Table S7), suggesting very minor, if any, genomic alterations in germ lines at the scale in this study. This was also demonstrated by an analysis using cnvPartition (a plug-in for copy number variation analysis of Genome Studio) (Figure S5 and Table S8).

**Supplementary Fig. S6:**
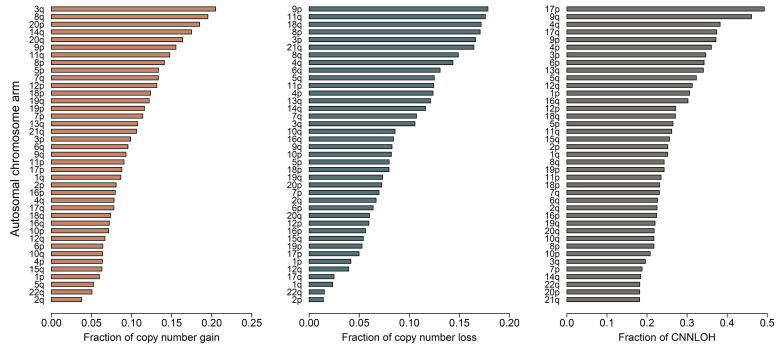
**Overview of genomic alteration fractions on chromosome arms across 55 ESCC samples**. CNG, copy number gain; CNL, copy number loss; CNNLOH, copy number neutral loss of heterozygosity.

Regarding the type of genomic alterations in ESCC, the median fraction of CNNLOH across the entire genome in all 55 samples was 0.29 (in the range 0–0.67), suggesting frequent occurrences of acquired uniparental disomy during mitosis. The median overall genome-wide fractions for copy number gain and loss were 0.09 (in the range of 0–0.36) and 0.05 (in the range of 6 × 10^−5^–0.38), respectively (Table S6). Among the 55 tumor samples examined, chromosome 3q harbored the largest fraction of copy number gain, whereas chromosome 9p had the largest fraction of copy number loss and chromosome 17p showed the largest fraction of CNNLOH (Table S9 and Figure S6).

### Both *TP53* mutations and CNAs point to cell cycle deregulation

**Figure 2:**
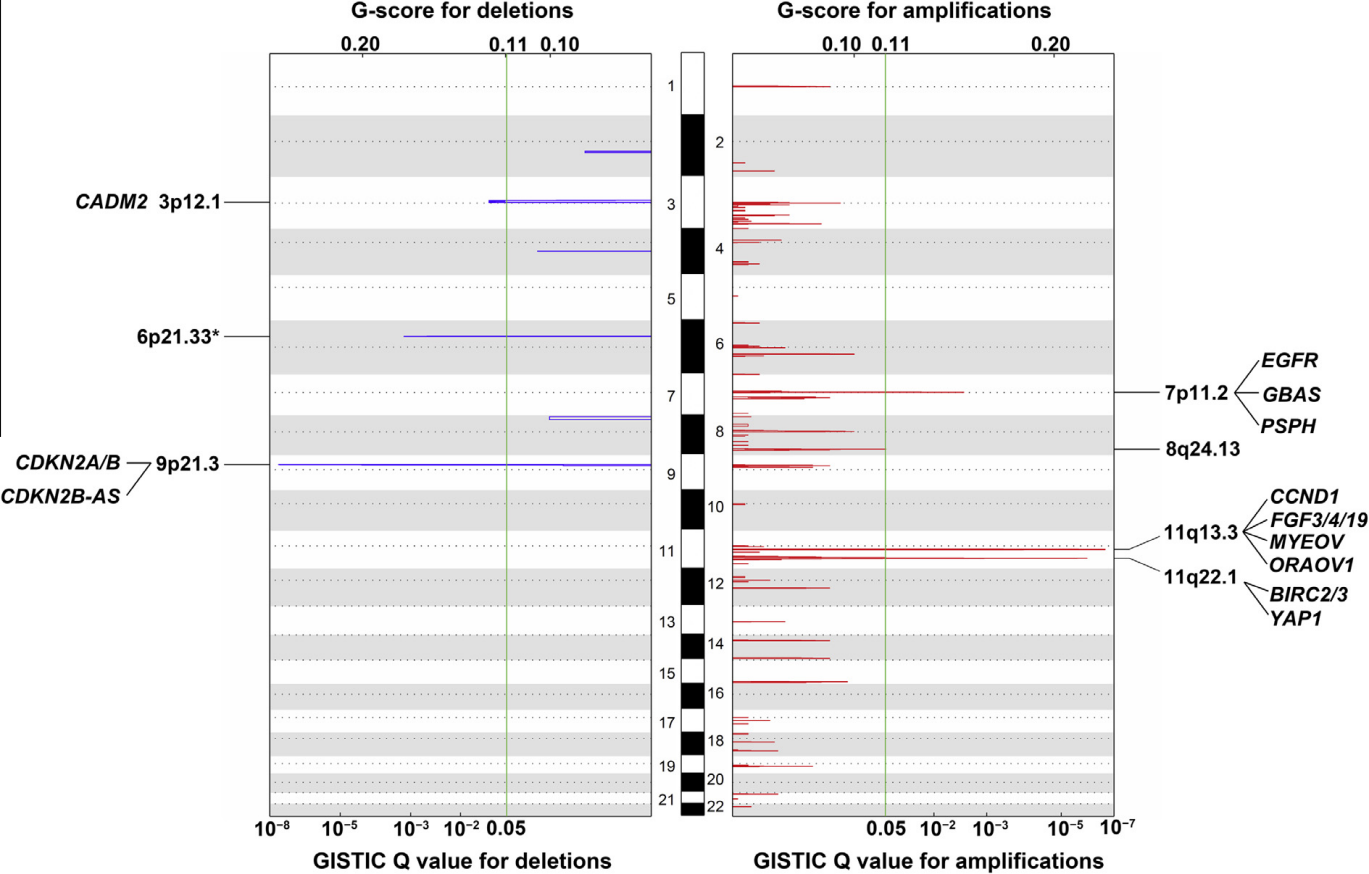
Profiling of genomic deletions and amplifications in 55 ESCC samples The log R ratios of SNPs were segmented by R‐GADA algorithm [60]. The peaks in red and blue represent the GISTIC Q value (bottom) and G-score (top) of amplified regions and deleted regions, respectively. The green vertical lines indicate the Q value considered as significant in the analysis (0.05). Across autosomes 1–22, six MCRs, including 2 deletions and 4 amplifications, were identified (*Q* < 0.05). Genes that have been reported to be related to either ESCC or other cancers in previous studies are listed in each MCR. The MCR in the HLA region of chromosome 6 (asterisk) was not included in this study due to its extremely high degree of polymorphism. Chromosomes are represented in white (odd-numbered chromosomes) and gray (even-numbered chromosomes) rectangles alternately (with heights in proportion to the lengths of the respective chromosomes). Positions for centromeres are indicated by the dotted lines. R, probe intensity in the SNP array; MCR, minimal common region; HLA, human leukocyte antigen.

Among the 55 tumor samples analyzed, we identified four significantly-amplified MCRs containing 34 known genes, as well as two deleted MCRs covering 19 genes (Figure 2, Table S10). Among them, MCRs on 11q13.3 and 9p21.3 were the most significantly amplified and deleted regions, respectively (*Q* = 2.41 × 10^−7^ and 2.76 × 10^−8^; 17/55 and 17/55, respectively). This resulted in the amplification of several oncogenes, including *CCND1*, *FGF3*, *FGF4*, and *FGF19*, and the deletion of the tumor suppressor genes *CDKN2A* and *CDKN2B*. In addition, the region with focal amplification in 11q22.1 (*Q* = 1.25 × 10^−6^, 9/55) contains several cancer-related genes, including *YAP1*, which has been reported in the liver and colorectal cancers [20–22], as well as *BIRC2* and *BIRC3*, which activate the NF-κB signaling pathway (KEGG pathway database, http://www.genome.jp/kegg/pathway.html). Additionally, *EGFR* was amplified in the MCR of 7p11.2, whereas *CADM2*, which has been identified as a tumor suppressor gene in prostate cancer [23,24], was deleted in the MCR of 3p12.1.

**Figure 3:**
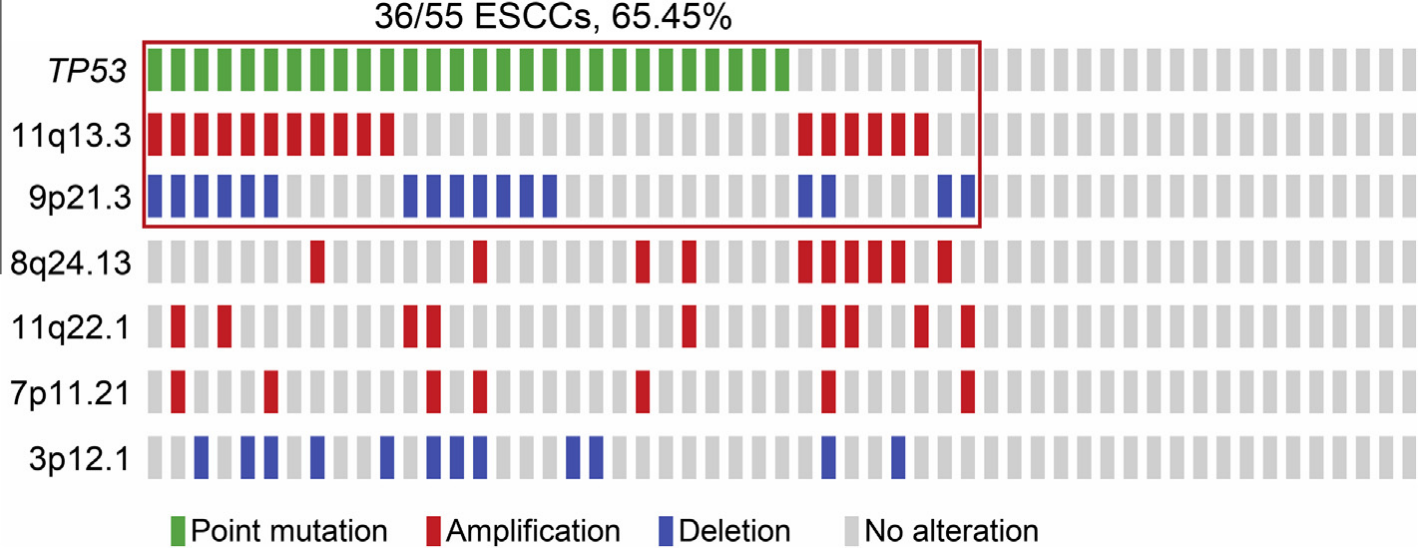
Occurrence of *TP53* mutations and CNAs of six MCRs in 55 samples Each solid bar in the same column represents a single case with color codes as green for point mutation, red for amplification, blue for deletion, and light gray for no alteration as indicated. The red box indicates 65% (36/55) of samples containing either the *TP53* nonsynonymous mutations (in 28 samples), the amplification of 11q13.3 containing *CCND1* (in 17 samples), or the deletion of 9p21.3 containing *CDKN2A/CDKN2B* (in 17 samples). CNA, copy number alteration.

**Figure 4:**
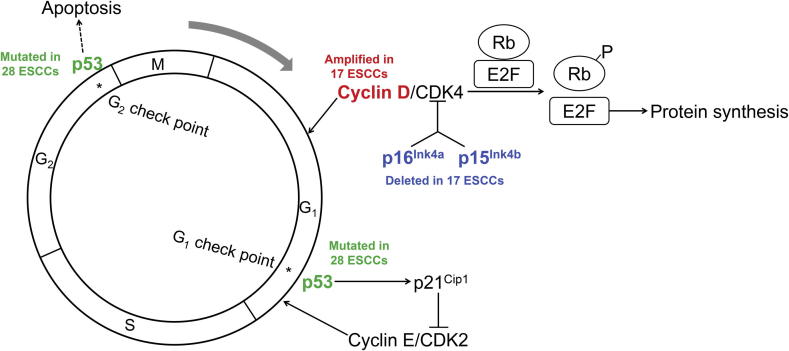
Genomic alterations point to cell cycle G1 deregulation Both *TP53* mutations and CNAs point to G1 deregulation in around two-thirds of ESCC tumor samples. Numbers of samples with *TP53* somatic mutation, focal amplification, and focal deletion are labeled in green, red, and blue, respectively.

Taken together, we saw a clear grouping in ESCC cases that were enrolled in this study (Figure 3). About 2/3 of the samples carried *TP53* somatic mutations or focal CNAs that make a large contribution to damaging cell cycle regulation. As demonstrated in Figure 4, the most affected nodes are at p16^INK4A^/p15^INK4B^, cyclin D1, and p53, which were caused by focal deletion of *CDKN2A*/*2B* (30.9%), focal amplification of *CCND1* (30.9%), and point mutations in *TP53* (50.9%), respectively. Among the 55 cases examined, 65.45% (36/55) of the patients carried at least one of these alterations. Considering the functions of p16^INK4A^ and cyclin D1 in G1 progression, as well as the role that p53 plays in mitotic check points, cell cycle regulation appears to be greatly disrupted in tumor cells of ESCC.

### ESCCs with *TP53* mutations are more genomically unstable

**Figure 5:**
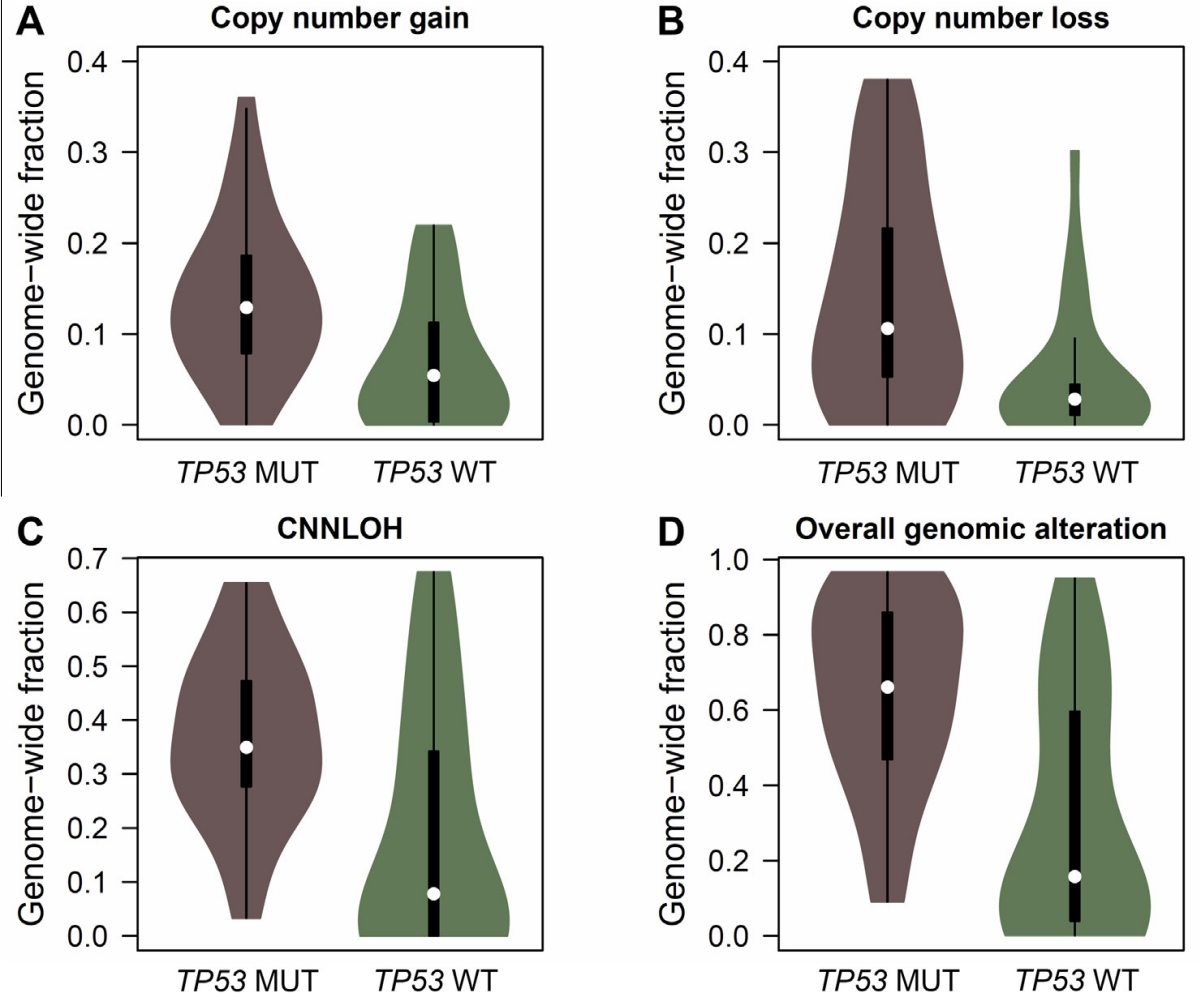
Association of *TP53* somatic mutations and genomic instability Violin plots show the genomic alterations including copy number gain (**A**, *P* = 1.18 × 10^−3^), copy number loss (**B**, *P* = 1.43 × 10^−3^), and CNNLOH (**C**, *P* = 9.65 × 10^−4^) in ESCC tumors with (*TP53* MUT) and without (*TP53* WT) *TP53* somatic mutations. The overall fraction of genomic alterations is shown in panel **D** (*P* = 1.10 × 10^−4^). CNNLOH, copy number neutral loss of heterozygosity. Mann–Whitney U test was performed to determine the significant differences.

We found a high correlation of *TP53* mutations with genome instability. As depicted in Figure 5, the median fractions of copy number gain, copy number loss, and CNNLOH in samples with *TP53* mutations were 0.13, 0.11, and 0.35, respectively. These values were significantly higher than those seen in samples with wild-type *TP53,* which were 0.05, 0.03, and 0.08, respectively (Mann–Whitney U test *P *= 1.18 × 10^−3^, 1.43 × 10^−3^, and 9.65 × 10^−4^, respectively). In particular, 75% of the patients with *TP53* mutations had severe genomic instability, while only 33% of the patients with wild-type *TP53* had severe genomic instability (*P* = 0.002).

### Chromothripsis likely occurs in one tumor sample

**Figure 6:**
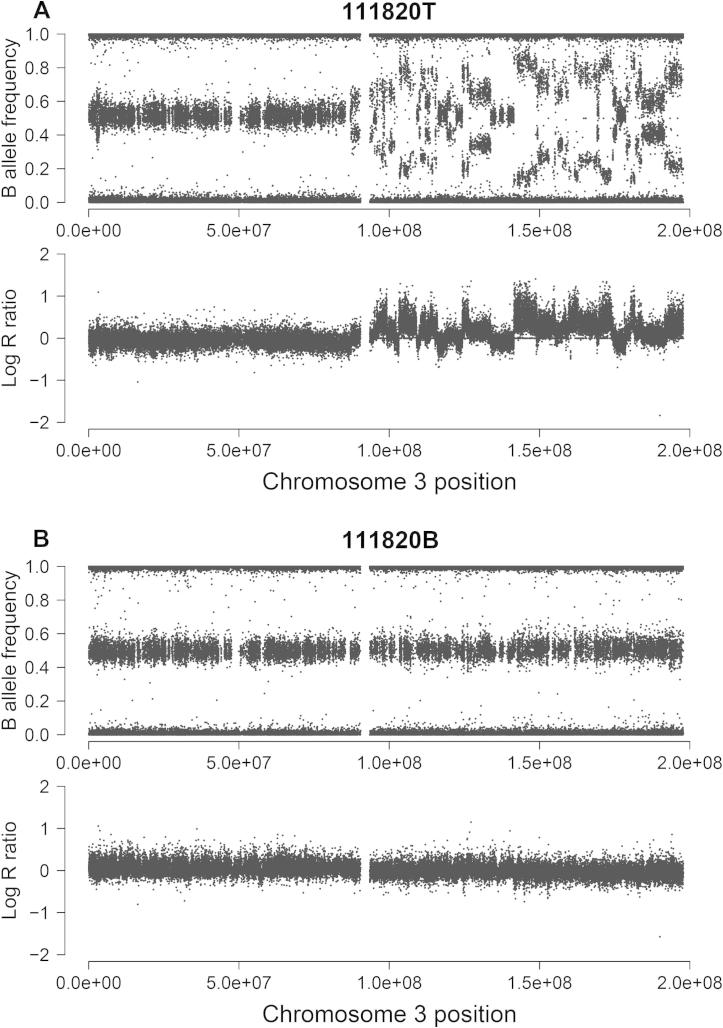
Occurrence of chromothripsis in one ESCC tumor sample The genomic evidence for chromothripsis in one ESCC case (patient ID 111820) is shown by comparison of tumor sample (**A**) with the matched blood sample (**B**). Both B allele frequency and log R ratio on the chromosome arm 3q of the tumor sample demonstrated numerous break points with limited copy number alterations.

Shattering of chromosomes or chromosome arms was reported in a fraction of cancer samples, which is believed to be the driver event in these cases [25]. In the SNP array screen of 55 tumor samples, 42 break points were observed on the chromosome arm 3q in one tumor sample (patient ID 111820; 1/55 or 1.8%), as demonstrated in Figure 6. This suggests the occurrence of a genomic instability-generating phenomenon known as chromothripsis, where tens to hundreds of chromosomal rearrangements occur in a “one-off” cellular event [25]. Currently, the clinical consequences of this low-prevalence genomic event in ESCC remain unknown due to the limited sample size in our study.

### Correlation of genomic alterations with clinical outcome

**Supplementary Fig. S7:**
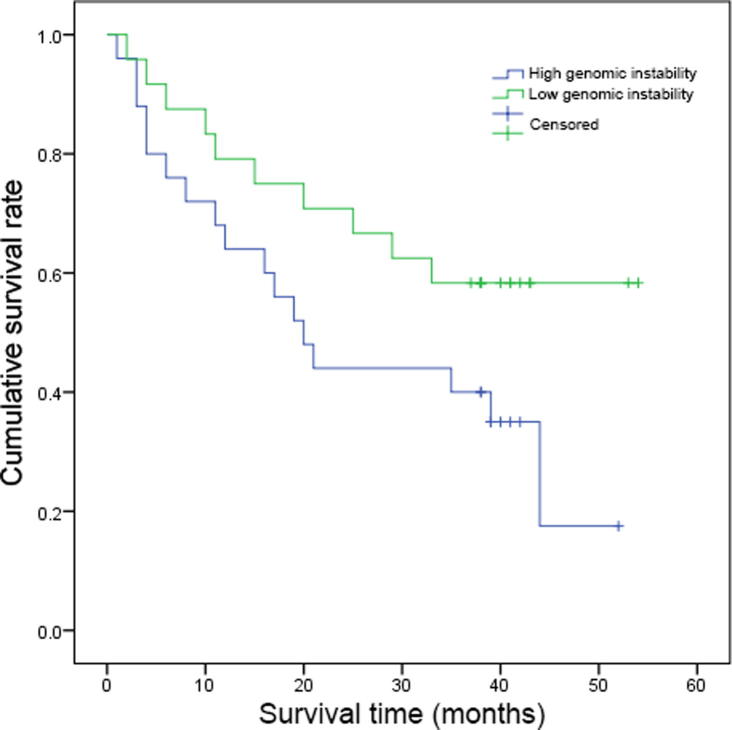
**Association between genomic instability level and patient survival rate**. Patients with higher degrees of genomic instability (⩾50% of the genome showing alterations, 25 patients) tended to have poorer post-surgery survival than those with a low degree of genomic alteration (<50% of the genome showed alterations, 24 patients), though the difference was not statistically significant (*P* = 0.083).

We also analyzed the association of genomic instability with clinical information (gender, age, cancer stage and differentiation, tobacco and alcohol consumption, and family cancer history) and survival status. Patients with severe genomic instability (⩾50% of the genome) had a higher percentage of lymph node metastasis than patients with a low (<50% of the genome) genomic instability (63.3% *vs.* 36.7%, *P* = 0.021, Table S11). In addition, patients with a higher degree of overall genomic instability showed poorer survival, although the difference was not statistically significant (*P* = 0.083, Figure S7). Moreover, among the six MCRs identified, three regions were significantly correlated with lymph node metastasis, including the amplification of 7p11.2 (*P* = 0.042), deletion of 3p12.1 (*P* = 0.030), and deletion of 9p21.3 (*P* = 0.033) (Figure 2; Table S12). On the other hand, although non-synonymous *TP53* mutations occurred more frequently, we observed no correlation between these mutations and clinical outcome (data not shown).

## Discussion

In this study, we performed exome sequencing on nine pairs of ESCC samples, followed by an analysis of structural alterations using SNP arrays. Due to the relatively low coverage of exome sequencing, we eliminated all of the false positive calls through Sequenom or Sanger sequencing verification. Although we could not avoid certain false negative calls, the very similar mutation rates of *TP53* in the discovery set (5/9) and validation set (23/46) indicate that our exome sequencing data are reliable. Moreover, we re-sequenced five exons from three frequently-mutated cancer genes, *AKT1*, *PIK3CA*, and *BRAF*, in an additional 120 ESCC samples. The inability to detect somatic mutations also suggests that high frequency mutations are seldom seen in ESCC other than in *TP53*. This is consistent with the findings of recent genomic studies of ESCC, in which *TP53* was found to be the most frequently mutated gene (>60% in all studies) [26–29]. Other potential driver genes defined by these studies mutated only in ∼20% samples at most, which were identified as singleton in our exome sequencing study, for instance somatic frameshift indel identified in *ADAM29* from patient 101919.

Exome sequencing showed limited resolution of structural alterations, as indicated by the allelic imbalance (Figure 1). Therefore, to characterize these alterations more precisely, we conducted a whole-genome SNP array using both the discovery sample set and an additional set of 46 tumors. To ensure detection of structural alterations in cancer cells, we set our criterion for a segment containing at least 50 continuous SNPs. In this way, we neglected smaller germline copy number variations. Using a scanning scale of roughly 150 kb (one SNP per 3 kb on average), we observed striking evidence of genomic instability (including copy number gain, loss, and neutral LOH) than that in the blood samples. As a result, our SNP array data support that the allelic imbalance in our exome sequencing data reveals valuable information regarding structural alterations.

### Deregulation of cell cycle, especially in G1 phase, plays an important role in ESCC

A number of *in vitr*o and *in vivo* experiments have demonstrated the key role of cell cycle deregulation in tumorigenesis [30–33]. Many cell cycle-related factors function as either tumor suppressors or oncogenes [34–36]. The most important molecule in this process is *TP53*, whose high mutation rate has been reported in various types of cancer (IARC *TP53* Database, http://p53.iarc.fr/). In this study, we showed strong new evidence for the old story of cell cycle deregulation in ESCC. Somatic mutations in *TP53* plus two MCRs (deletion of *CDKN2A*/*2B* and amplification of *CCND1*) point primarily to cell cycle deregulation in over 65% of the cases examined. Therefore, *TP53* mutations, which disrupt cell cycle check points at the G1/S and G2/M transitions, and the amplification of an oncogene (*CCND1*) or the deletion of suppressor genes (*CDKN2A*/*2B*) strongly suggest that the disruption of G1 control is a key event in the development of ESCC (Figure 4). Although the high rate of *TP53* mutation and the complex DNA alterations have been observed in ESCC and other cancers [37–41], to our knowledge this is the first analysis on a large sample size that pinpoints the high frequency of G1 control deregulation caused by genomic alterations in ESCC.

### *TP53* somatic mutations are correlated with severe genomic instability

We observed a very high correlation between somatic mutations in *TP53* and genomic instability, which has been observed in many previous studies [42–45]. In tumors with a genomic instability fraction <0.10, only 1 out of 14 samples had a mutated *TP53*. Conversely, in samples with fraction >0.90 for structural alteration, six out of seven samples had *TP53* mutations. One assumption is that the *TP53* mutations and genomic alteration may occur independently and that *TP53* mutations result in a failure to undergo apoptosis in cells carrying genomic abnormalities [46–48]. On the other hand, *TP53* has also been proposed to be the “genome guardian” and mutations in this gene may therefore directly cause other genomic alterations [33,49]. In our ESCC cases, despite the high mutation rate of *TP53*, we found no significant association between *TP53* mutation and tumor stage, whereas genomic instability correlated with lymph node metastasis. Therefore, we speculate that the *TP53* mutation may occur early during ESCC development, and one of the consequences is genome instability, as suggested by many previous studies [50,51].

### Diverse genomic patterns reveal other potential drivers in ESCC development

In this study, we found 19 samples without any alteration in cell cycle regulation. Based on their genome instability status, these cases can further be classified into three types. Two of the 19 samples had severe genomic instability (>0.5) but contained none of the six MCRs that were identified in other individuals. Both samples shared focal amplifications at 8p11.21, which encompasses *IKBKB*, a node in the NF-κB pathway. Another nine samples had medium genome-wide instability with alteration fractions of 0.05–0.35. However, we failed to identify any major common patterns of somatic mutations or structural alterations in these nine tumors. More interestingly, the remaining 8 samples showed extremely low fractions of genomic instabilities (<0.05). Three out of these 8 samples were analyzed by exome sequencing with no somatic mutations detected. Although we cannot rule out the possibility of a few false negatives, these examples suggest a special category of ESCC that carries low numbers of structural variations. It is therefore, of particular interest to further study ESCC that has a relatively stable genome. Furthermore, these findings may provide fundamental insights into the molecular classification of ESCC.

### Other cancer-related mutations and pathway alterations occur in ESCC

In addition to cell cycle-related copy number gains, several other genes were also amplified by the 11q13.3 focal change, such as *FGF3/4/19* and *CTTN*. *FGF3/4/19* gene cluster encodes fibroblast growth factor 3, 4, and 19, members of the FGF family. Considering their activities in the MAPK and PI3K-Akt signaling pathways, these amplified factors may also be involved in mitogenic and cell survival in ESCC. A few over-expressed genes in cancer are also located in the amplified region of 11q13.3, including *MYEOV* (myeloma over expressed) [52], *ORAOV1* (oral cancer over expressed gene 1) [53], *ANO1* in head and neck squamous cell carcinoma [54], and *CTTN* in breast cancer and squamous cell carcinomas of the head and neck [55,56]. Therefore, although *CCND1* was considered as the major player in this MCR, the amplification of other genes may also contribute to ESCC development.

Furthermore, despite the fact that no common point mutations other than *TP53* were found in our study, we observed one recurrently-mutated gene, *FBXL4,* in both the discovery (2/9) and validation sets (2/120). The mutation rate was 3.1% for this gene encoding F-box protein, which is one subunit of the ubiquitin protein ligase complex, implying that there is disruption in the ubiquitination in some ESCC samples. These observations call for further investigation of the ubiquitin-related protein degradation pathway in ESCC patients.

### Clinical significance of our findings

In this study, we observed an association of overall tumor genomic instability and clinical outcome. In particular, we discovered that three MCRs, amplification of 7p11.2, deletion of 3p12.1, and deletion of 9p21.3, correlated with lymph node metastasis. Several known cancer-related genes, including *EGFR* and *CDKN2A/2B,* are harbored in these regions. Additionally, the function of a few other genes, such as *GBE1* and *CADM2* in 3p12.1, has not been intensively studied in cancers and thus deserve further investigation. These findings suggest that carriers of these MCRs may have poor disease progression and therefore, detection of these alterations may be helpful in future disease monitoring.

Furthermore, as the heterogeneity has been widely accepted as a common feature in cancer, a clinic challenge is the therapeutic strategy regarding how to deliver proper and individualized treatments. We observed a clear classification in ESCC cases as the G1 deregulation occurred in 2/3 of the cases examined. Such a high deregulation frequency in certain pathways provides promising targeting strategies for future individualized diagnoses and therapeutic development. In particular, the CDK inhibitors that target early G1 phases may be beneficial for this group of patients.

## Conclusions

In this study, we found a ∼50% prevalence of *TP53* mutations in ESCC, and the somatic mutation of *TP53* is highly correlated with genomic instability. Three MCRs are associated with lymph node metastasis. The amplification of *CCND1* and the deletion of *CDKN2A/2B,* together with *TP53* mutations, may play pivotal roles in ESCC by deregulating G1 cell cycle signaling, which classify this cancer into different groups.

## Materials and methods

### Sample collection

All ESCC patients enrolled in this study were diagnosed at the Anyang Cancer Hospital (Henan Province, China) in 2007–2009. Tumor specimens and paired blood samples were collected from ESCC patients. Written informed consent was obtained before sample collection. Tumor specimens from ESCC cases were stored at −70 °C immediately following collection. Genomic DNA was purified from tumor samples using a Biomek 3000 automated workstation with a E.Z.N.A Mag-Bind Tissue DNA Kit (Omega Bio-Tek, Norcross, GA, USA), whereas DNA was extracted from blood samples using a Whole Blood DNA Extraction Kit (BioTeke, Beijing, China). DNA quality and quantity were determined using a NanoDrop 1000 (Thermo Scientific, Wilmington, DE, USA).

Demographic data and patient information, including age, gender, alcohol and tobacco consumption history, and family history of cancer, were obtained from patients’ medical records, as listed in Table S1. Tumor type, tumor cell content, histological classification, and cancer grade and stage were reviewed by two pathologists independently. The content of tumor cells in each sample was over 70%, as shown by hematoxylin and eosin staining (Figure S1). The clinical information for the other 46 tumor samples subjected to SNP array genotyping is shown in Table S1. The tumor samples used for SNP array genotyping and mutational rate validation of candidate genes are shown in Figure S2. This study was approved by the Institutional Review Board of the School of Oncology, Peking University, China.

### Exome sequencing data processing

Exome sequencing was performed for nine pairs of ESCC tumor samples and the matched blood samples. Genomic DNA libraries were prepared using the Pair-End Genomic DNA Sample Prep Kit (Illumina, San Diego, CA, USA). The genomic DNA was sequenced using the Illumina Genome Analyzer IIx for 75-cycles at BGI, Shenzhen. All original sequencing tags were converted to the FASTAQ format. To increase the accuracy and specificity of read mapping and mutation identification, several pre-processing filters were applied to the raw sequencing tags. For each sequencing tag, if two adjacent bases had a Phred quality score <20, then these two bases and the following bases were trimmed from the tag, and tags shorter than 35 nt were excluded.

### Identification and annotation of the somatic mutations

After the pre-processing quality control, tags were aligned to the hg18 version of the human genome using the BWA (Burrows-Wheeler Aligner, version 0.5.8) [57]. Single nucleotide substitutions and small insertion/deletions (indels) were identified using SAMtools (Utilities for the Sequence Alignment/Map format) [58]. Under this filtration, the average number of variants called per sample was ∼15,957 (12,942–21,892), and the transition/transversion (Ti/Tv) ratio was 2.74 (on average, ranging 2.40–2.95 for all samples).

In order to identify and further increase the specificity of somatic mutation calls, we applied the following post-processing filters: (1) only loci with ⩾10× coverage in both tumor and normal samples were used for variant calling; (2) at least 20% of mutant alleles in reads from tumor samples had a Phred quality score ⩾20; and (3) no mutant alleles were detected in reads from the blood samples.

All NSSMs identified by exome sequencing were subjected to Sequenom MassARRAY (Sequenom, San Diego, CA, USA) or conventional Sanger sequencing for validation. Genomic positions and flanking sequences for all SNVs were retrieved using the hg18 version of the human genome and the University of California Santa Cruz (UCSC) genome annotation database. For Sequenom MassARRAY, PCR and MassEXTEND® primers for multiplexed assays were designed using the Sequenom MassARRAY Assay Design 3.1 software. The allele-specific extension products of different masses were quantitatively analyzed using MALDI-TOF mass spectrometry. Mutation calls were determined using a MassARRAY Typer 4.0 Analyzer, according to the manufacturer’s specifications. For Sanger sequencing, PCR primers were designed using Primer Premier 5.0 (PREMIER Biosoft, Palo Alto, CA, USA). The gene ontology (GO) cluster analysis of all mutated genes was done using the Functional Annotation Tool of the Database for Annotation, Visualization and Integrated Discovery (DAVID) [59].

### Survey of allelic imbalance

The minor allele fraction (MAF) of each informative SNP was calculated as a measure of allelic imbalance as shown below.

$$\text{MAF}=\frac{\text{Number of reads containing minor allele}}{\text{Number of reads containing major allele}}$$

An informative SNP was defined as a known heterozygous SNP at target regions with the coverage of at least 10 distinct reads in both tumor and blood samples. A difference of MAF in blood (MAF_B_) and tumor (MAF_T_) samples of more than 0.1 was considered to be significant (MAF_B_ − MAF_T_ ⩾ 0.1). Window sliding was carried out to estimate the size of the altered regions. With a window size of 50 SNPs, a window was considered to be a region undergoing somatic alteration if more than 70% of the SNPs had a significantly-different MAF in the tumor and blood samples.

### Whole-genome SNP genotyping and data analysis

Whole-genome SNP array for 55 ESCC samples was performed on Illumina Human OmniZhongHua BeadChips (Illumina, San Diego, CA, USA). Raw intensity values were processed to obtain a normalized B allele frequency (BAF) and a Log R ratio (LRR) for each probe using the Genome Studio Software V2011.1. LRR values were segmented with the Genome Alteration Detection Analysis (GADA) [60] using parameters of T > 10 and segment lengths containing ⩾50 continuous probes. For LOH analysis, the aforementioned window sliding approach was used with a window size of 100 informative SNPs. A window was considered a LOH, if more than 80% of the SNPs had a MAF ⩽ 0.9. A segment was defined either as normal or as having one of 5 types of alteration status based on the following criteria: (1) normal, |LRR| < 0.075 and retaining heterozygosity; (2) gain, LRR ⩾ 0.075; (3) loss, LRR ⩽ −0.075; (4) CNNLOH, |LRR| < 0.075; (5) amplification, LRR ⩾ 0.15; and (6) deletion, LRR ⩽ −0.15.

To survey for genome instability, the genomic fractions of copy number gain, loss, and CNNLOH were estimated by dividing the number of SNPs undergoing a specific alteration by the total number of SNPs present in the respective chromosome or in the respective sample. To identify common regions with copy number gain and loss across samples, the Genomic Identification of Significant Targets in Cancer (GISTIC) algorithm was utilized [61]. Thresholds of LRR were set as 0.15 and −0.15 to allow GISTIC to identify amplifications and deletions, respectively. Q values of MCRs < 0.05 were defined as significant, and 0.99 was used as the confidence level to determine the region that contained potential driver genes.

### Statistical analysis

All statistical analysis was performed using R version 3.1.2 or SPSS version 19.0 (IBM, Armonk, New York, USA). The association of overall survival with genomic instability status was evaluated with Kaplan–Meier curves, and differences were tested using the log-rank test. Difference was considered significant with *P* ⩽ 0.05.

## Authors’ contributions

CZ, YK, and HC conceived the study, supervised all aspects of the work, and co-wrote the manuscript. QW and JB performed the analysis and interpretation of sequencing data and SNP array data, participated in majority of the experiments mentioned in the paper, and co-wrote the manuscript. AA, YL, KG, JL, FL, and YP performed sample collection and preparation, somatic mutation validation, and PCR-Sanger sequencing of candidate genes. SL and WS performed SNP genotyping. WC participated in the data analysis of exome sequencing and SNP genotyping. HY, CL, LZ, and RX performed sample collection. All authors read and approved the final manuscript.

## Competing interests

The authors have declared no competing interests.

## Supplementary material

Supplementary Table S1 **Clinicopathologic information of ESCC patients subjected to exome sequencing and SNP array genotyping**.

Supplementary Table S10 **Regions of focal amplification and deletion and the genes harbored**.

Supplementary Table S11 **Correlation between genomic instability and clinical data**.

Supplementary Table S12 **Minimal common regions associated with lymph node metastases**.

Supplementary Table S2 **The average read depths and fraction of coverage under different read depths of exome sequencing for each sample**.

Supplementary Table S3 **Characterization of non-synonymous somatic mutations and indels identified in the nine patients subjected to exome sequencing**.

Supplementary Table S4 ***TP53* mutations identified in 23/46 samples by Sanger sequencing**.

Supplementary Table S5 **Top five GO biological processes enriched**.

Supplementary Table S6 **Fractions of genomic alterations in 55 ESCCs detected by whole-genome SNP array**.

Supplementary Table S7 **Fractions of genomic alterations in nine blood samples from ESCC patients detected by whole-genome SNP array**.

Supplementary Table S8 **Fractions of genomic alterations in blood samples detected using cnvPartition**.

Supplementary Table S9 **Fractions of genomic alterations on different autosomal arms in 55 ESCC tumor samples**.
