# Supplementary material for: An Old Story Retold: Loss of G1 Control Defines A Distinct Genomic Subtype of Esophageal Squamous Cell Carcinoma
Source: Genomics Proteomics Bioinformatics. 2015 Sep 16;13(4):258–70. doi: 10.1016/j.gpb.2015.06.003 (PMC4610972; doi:10.1016/j.gpb.2015.06.003)
Supplement: Supplementary Table S1 — Clinicopathologic information of ESCC patients subjected to exome sequencing and SNP array genotyping. [file mmc1.rtf]

Table S1  Clinicopathologic information of ESCC patients subjected to exome sequencing and SNP array genotyping
Patient ID	Age	Gender	Grade	T 	N	Tobacco	Alcohol	Family cancer history	
99648*	46	M	3	3	1	Yes	Yes	Yes	
100036*	49	F	3	3	0	No	No	No	
101105*	64	F	3	2	0	No	No	No	
101506*	56	F	2	2	0	No	No	No	
101795*	60	M	2	3	2	No	No	No	
101815*	60	F	1	3	2	No	No	No	
101919*	65	M	3	3	1	No	No	No	
102995*	64	F	3	2	0	No	No	Yes	
103048*	60	M	3	3	3	No	No	Yes	
107859	64	M	3	2	0	Yes	Yes	No	
108073	56	M	3	3	2	Yes	No	Yes	
108607	78	M	2	3	0	Yes	Yes	No	
108736	74	M	2	3	0	Yes	No	No	
108932	74	F	2	2	0	No	No	No	
108960	58	M	3	2	2	No	No	Yes	
108987	54	F	2	3	1	No	No	No	
109121	71	F	2	3	0	No	No	No	
109382	63	M	1	2	0	No	No	Yes	
109554	67	M	2	2	0	No	No	No	
109596	52	M	3	3	0	Yes	Yes	No	
110165	61	M	3	3	1	Yes	No	Yes	
110197	50	M	3	2	1	Yes	No	No	
110269	62	F	3	1	0	No	No	Yes	
110270	59	F	3	2	1	No	No	No	
110274	63	M	3	2	0	No	No	No	
110390	64	F	2	3	0	No	No	No	
110440	56	M	2	3	1	No	No	No	
110850	59	M	2	3	0	Yes	No	No	
110852	61	M	2	2	0	Yes	No	No	
110855	61	M	2	3	0	Yes	No	No	
110892	76	M	3	3	2	Yes	Yes	Yes	
110957	60	F	2	2	2	Yes	No	No	
111531	67	M	2	3	1	No	No	No	
111603	69	F	2	3	0	No	No	Yes	
111667	68	M	2	3	1	Yes	Yes	No	
111726	48	F	2	2	0	No	No	No	
111820	56	M	2	2	0	No	No	Yes	
111822	69	M	3	3	1	No	No	No	
111898	59	M	3	2	1	Yes	No	Yes	
111926	69	M	2	3	0	Yes	Yes	Yes	
111930	62	F	2	3	1	No	No	Yes	
111944	64	F	2	2	2	No	No	Yes	
111954	63	M	2	2	3	No	No	No	
111958	59	F	2	2	2	No	No	No	
111959	70	M	3	3	1	Yes	Yes	No	
112053	40	M	3	3	0	Yes	Yes	Yes	
112077	53	M	2	3	0	Yes	No	No	
112282	76	M	2	2	1	Yes	Yes	No	
112325	69	F	2	2	0	No	No	No	
112400	70	M	3	2	1	Yes	No	Yes	
112402	64	M	3	3	0	Yes	No	No	
112552	62	M	2	3	1	Yes	Yes	No	
112644	65	M	3	3	0	Yes	No	Yes	
112648	52	M	2	3	0	No	No	No	
112887	73	F	2	3	2	No	No	Yes	
Note: * indicates the nine patients with tumor and blood samples subjected to exome sequencing. All patients were randomly selected from samples collected at Anyang Cancer Hospital from 2007 to 2009, as described in Materials and methods. Grade indicates degree of tumor differentiation (1, high; 2, middle, and 3, low); T indicates the size of the original (primary) tumor and whether invasion into nearby tissues occurred; and N indicates the extent of involvement of nearby (regional) lymph nodes. M, male; F, female.
