# Supplementary material for: An Old Story Retold: Loss of G1 Control Defines A Distinct Genomic Subtype of Esophageal Squamous Cell Carcinoma
Source: Genomics Proteomics Bioinformatics. 2015 Sep 16;13(4):258–70. doi: 10.1016/j.gpb.2015.06.003 (PMC4610972; doi:10.1016/j.gpb.2015.06.003)
Supplement: Supplementary Table S10 — Regions of focal amplification and deletion and the genes harbored. [file mmc10.rtf]

Table S10  Regions of focal amplification and deletion and the genes harbored
Cytoband	Region	Type of alternation	Genes harbored 	GISTIC Q value	No. of cases	
9p21.3	21865843 – 22451030	Deletion	CDKN2A, CDKN2B, C9orf53, CDKN2B-AS1	2.76E£­08	17	
11q13.3	69164141 – 70295760	Amplification	hsa-mir-548k, CCND1, CTTN, FGF3, FGF4, PPF1A1, FADD, FGF19, MYEOV, ANO1, ORAOV1, MIR548K	2.41E£­07	17	
3p12.1	74565111 – 86989133	Deletion	hsa-mir4273, hsa-mir-1324, GBE1, ROBO1, ROBO2, CADM2, LOC401074, LOC440970, FLJ20518,  FAM86DP, ZNF717, FRG2C, MIR1324, MIR4273, MIR4444-1	0.028	12	
8q24.13	127012373 – 127308156	Amplification	LOC100130231	0.049	11	
11q22.1	101605467 – 102364348	Amplification	BIRC2, BIRC3, YAP1, KIAA137, C11orf70, TMEM123, ANGPT5	1.25E£­06	9	
7p11.2	55023402 – 56206360	Amplification	CCT6A, EGFR, GBAS, PHKG1, PSPH, SUMF2, CHCDD2, MRPS1, LANCL2, VOPP1, SEPT14, ZNF713, FKBP9L, LOC389493, SNORA15	0.0029	7	
