# Supplementary material for: An Old Story Retold: Loss of G1 Control Defines A Distinct Genomic Subtype of Esophageal Squamous Cell Carcinoma
Source: Genomics Proteomics Bioinformatics. 2015 Sep 16;13(4):258–70. doi: 10.1016/j.gpb.2015.06.003 (PMC4610972; doi:10.1016/j.gpb.2015.06.003)
Supplement: Supplementary Table S11 — Correlation between genomic instability and clinical data. [file mmc11.rtf]

Table S11  Correlation between genomic instability and clinical data
Clinical data	Degree of instabilities	P value	
	Low (25 in total)
No. of patients (%)	High  (30 in total)
No. of patients (%)		
Tumor stage				
    T1	0 (0)	1 (3.3%)	0.881	
    T2	11 (44.0%)	11 (36.7%)		
    T3	14 (56.0%)	18 (60.0%)		
Lymph node metastasis				
    N0	17 (68.0%)	11 (36.7%)	0.021	
    N1	8 (32.0%)	19 (63.3%)		
Differentiation grade				
    High (G1)	1 (4.0%)	1 (3.3%)	0.94	
    Middle (G2)	13 (52.0%)	17 (56.7%)		
    Low (G3)	11 (44.0%)	12 (40.0%)		
Age				
    > 60 years	16 (48.5%)	17 (51.5%)	0.58	
    ≤ 60 years	9 (40.9%)	13 (59.1%)		
Gender				
    Male	8 (42.1%)	11 (57.9%)	0.717	
    Female	17 (47.2%)	19 (52.8%)		
Tobacco consumption 				
    Yes	11 (44.0%)	20 (66.7%)	0.091	
    No	14 (56.0%)	10 (33.3%)		
Alcohol consumption				
    Yes	19 (76.0%)	25 (83.3%)	0.498	
    No	6 (24.0%)	5 (16.7%)		
Family cancer history				
    Yes	17 (68.0%)	19 (63.3%)	0.717	
    No	8 (32.0%)	11 (36.7%)		
Note: Low and high genomic instability levels were defined as having < 50% and ≥ 50% genome-wide fraction of alterations, respectively. N0 and N1 indicate the absence and presence of lymph node metastasis, respectively.
