# Supplementary material for: An Old Story Retold: Loss of G1 Control Defines A Distinct Genomic Subtype of Esophageal Squamous Cell Carcinoma
Source: Genomics Proteomics Bioinformatics. 2015 Sep 16;13(4):258–70. doi: 10.1016/j.gpb.2015.06.003 (PMC4610972; doi:10.1016/j.gpb.2015.06.003)
Supplement: Supplementary Table S12 — Minimal common regions associated with lymph node metastases. [file mmc12.rtf]

Table S12  Minimal common regions associated with lymph node metastases
Region	Genomic alteration	Lymph node metastasis status	P value	
		N1 (27 in total)
No. of patients (%)	N0  (28 in total)
No. of patients (%)		
9p21.3	Deletion	12 (70.6%)	5 (29.4%)	0.033	
	Normal	15 (39.5%)	23 (60.5%)		
7p11.2	Amplification	6 (85.7%)	1 (14.3%)	0.030	
	Normal	21 (43.8%)	27 (56.3%)		
3p12.1	Deletion	9 (75.0%)	3 (25.0%)	0.042	
	Normal	18 (41.9%)	25 (58.1%)	¡¡	
Note: Statistical analysis was performed using Fisher's exact test. 
