# Supplementary material for: An Old Story Retold: Loss of G1 Control Defines A Distinct Genomic Subtype of Esophageal Squamous Cell Carcinoma
Source: Genomics Proteomics Bioinformatics. 2015 Sep 16;13(4):258–70. doi: 10.1016/j.gpb.2015.06.003 (PMC4610972; doi:10.1016/j.gpb.2015.06.003)
Supplement: Supplementary Table S2 — The average read depths and fraction of coverage under different read depths of exome sequencing for each sample. [file mmc2.rtf]

Table S2  The average read depths and fraction of coverage under different read depths of exome sequencing for each sample
Sample ID	Average read depth	Targeted region coverage (%) at different read depths	
		£¾1 ×	£¾5 ×	£¾10 ×	£¾15 ×	
99648B	28.1 	94	73	63	55	
99648T	19.6 	93	64	52	42	
100036B	22.4 	94	78	65	51	
100036T	31.3 	94	74	65	57	
101105B	35.0 	94	78	69	62	
101105T	30.3 	94	73	63	55	
101506B	26.1 	93	70	60	52	
101506T	35.9 	94	83	77	71	
101795B	34.2 	95	81	71	63	
101795T	27.7 	94	70	60	52	
101815B	33.0 	94	80	71	63	
101815T	33.9 	95	79	69	61	
101919B	32.2 	94	75	66	58	
101919T	49.1 	95	92	90	86	
102995B	37.0 	95	82	74	66	
102995T	25.9 	93	70	59	51	
103048B	32.0 	95	79	69	60	
103048T	31.0 	94	76	66	58	
Note: B and T in the sample ID indicate blood and tumor samples, respectively.
