# Supplementary material for: An Old Story Retold: Loss of G1 Control Defines A Distinct Genomic Subtype of Esophageal Squamous Cell Carcinoma
Source: Genomics Proteomics Bioinformatics. 2015 Sep 16;13(4):258–70. doi: 10.1016/j.gpb.2015.06.003 (PMC4610972; doi:10.1016/j.gpb.2015.06.003)
Supplement: Supplementary Table S3 — Characterization of non-synonymous somatic mutations and indels identified in the nine patients subjected to exome sequencing. [file mmc3.rtf]

Table S3  Characterization of non-synonymous somatic mutations and indels identified in the nine patients subjected to exome sequencing
Patient ID	Gene	Chr	Position	Reference
allele	Genotype
in tumor 	AA change	rs ID	SIFT	PolyPhen2	
101105	KIF2C	1	44993034	G	G/T	W176L	N/A	Damaging	Benign	
	WNT2B	1	112864482	G	G/T	C243F	N/A	Damaging	Probably damaging	
	DCST1	1	153280870	#	+AGTG	--	N/A	Frameshift	N/A	
	PDE1A	2	182813088	#	£­C	--	N/A	Frameshift	N/A	
	SPAG16	2	214983231	G	A/G	G615R	N/A	Damaging	Probably damaging	
	CHL1	3	377045	A	A/C	Y415S	N/A	Tolerated	Probably damaging	
	PLXNB1	3	48431704	#	£­T	--	N/A	Frameshift	N/A	
	TBC1D22B	6	37392513	C	C/T	R408C	N/A	Damaging	Probably damaging	
	FBXL4	6	99428892	T	C/T	K617E	N/A	Damaging	Possibly damaging	
	SMARCD3	7	150567144	G	G/T	S477*	rs199976092	N/A	N/A	
	OR5D13	11	55297653	C	A/C	S55*	N/A	N/A	N/A	
	UBN1	16	4865019	C	A/C	S869R	rs199975020	Damaging	Possibly damaging	
	TP53	17	7517820	#	+TCC	--	N/A	AA insertion	N/A	
	C17orf48	17	10549103	C	A/C	Y45*	N/A	N/A	N/A	
	ZNF490	19	12553327	C	C/T	E188K	N/A	Tolerated	Benign	
	LILRB4	19	59867243	G	A/G	R97H	rs199821178	Tolerated	Benign	
	SYCP2	20	57889432	T	C/T	K919R	N/A	Tolerated	Benign	
	SH3KBP1	X	19474088	G	A/G	S462L	N/A	Damaging	Probably damaging	
	SLITRK2	X	144712059	G	A/G	D142N	N/A	Damaging	Probably damaging	
101506	C1QB	1	22860233	C	C/T	R177*	N/A	N/A	N/A	
	ZNF496	1	245539663	C	C/T	D285N	N/A	Damaging	Possibly damaging	
	CAPN10	2	241205087	g	C/G	*140S	N/A	Tolerated	N/A	
	NEK10	3	27272832	G	G/T	N80K	N/A	Tolerated	Benign	
	THAP9	4	84058451	C	C/T	H688Y	N/A	Damaging	Benign	
	ANK2	4	114494093	G	A/G	E1624K	N/A	Tolerated	Possibly damaging	
	FBXW7	4	153466625	T	C/T	R463G	N/A	Damaging	Probably damaging	
	CAST	5	96126729	C	C/G	L300V	N/A	Damaging	Probably damaging	
	FBXL4	6	99481098	G	A/G	S163F	N/A	Tolerated	Possibly damaging	
	RBM16	6	155150753	C	C/G	Q76E	N/A	Damaging	Possibly damaging	
	XRCC2	7	151977018	C	C/T	G162E	N/A	Damaging	Possibly damaging	
	WRN	8	31045318	#	+G	--	N/A	Frameshift	N/A	
	C8orf45	8	67971736	A	A/T	E533D	N/A	Tolerated	Possibly damaging	
	SORBS1	10	97091339	A	A/C	V586G	N/A	Damaging	Possibly damaging	
	PNLIPRP1	10	118350685	G	G/T	E349*	N/A	N/A	N/A	
	OR51B6	11	5329336	C	C/T	S8F	N/A	Tolerated	Benign	
	GPR83	11	93753262	C	C/T	A325T	N/A	Tolerated	Possibly damaging	
	CCDC82	11	95756145	a	A/T	N309K	N/A	Tolerated	Possibly damaging	
	DDX10	11	108099253	C	C/G	L607V	N/A	Tolerated	Benign	
	UTP14C	13	51500464	G	C/G	E406Q	N/A	Damaging	Probably damaging	
	MYH7	14	22963056	C	C/T	R941H	N/A	Damaging	Probably damaging	
	PCNX	14	70498766	A	A/T	R145*	N/A	N/A	N/A	
	ZNF75A	16	3307384	G	C/G	K135N	N/A	Damaging	Probably damaging	
	TP53	17	7520102	G	A/G	Q104*	N/A	N/A	N/A	
	CHD3	17	7750630	G	A/G	E1465K	N/A	Damaging	Probably damaging	
	ZNF587	19	63062553	G	A/G	E321K	rs200133587	Damaging	Probably damaging	
	C20orf27	20	3684173	C	C/T	E107K	rs138776644	Tolerated	Possibly damaging	
	ZNF334	20	44566676	G	G/T	Q50K	N/A	Damaging	Possibly damaging	
	C21orf123	21	45665826	G	A/G	A97V	rs2838920	Not scored	N/A	
	DMD	X	32290930	A	A/C	L1741V	N/A	Damaging	Probably damaging	
	FAM47C	X	36939501	G	A/G	E1033K	N/A	Tolerated	Benign	
	PHF16	X	46777977	G	A/G	G233D	N/A	Damaging	Probably damaging	
	ZCCHC5	X	77799505	G	A/G	L357F	N/A	Tolerated	Benign	
	MORC4	X	106072865	G	A/G	Q638*	N/A	N/A	N/A	
	KLHL13	X	116917120	C	C/T	A583T	rs151170440	Tolerated	Possibly damaging	
	AFF2	X	147551416	G	A/G	W155*	N/A	N/A	N/A	
101795	CR2	1	205717997	C	C/T	S930L	rs138062179	Tolerated	Possibly damaging	
	ZNF451	6	57121318	#	+A	--	N/A	Frameshift	N/A	
	RP1	8	55699943	#	£­GAT	--	N/A	AA deletion	N/A	
	KIAA1128	10	86121330	C	C/T	S181L	N/A	Tolerated	Possibly damaging	
	RASGRP2	11	64263481	A	A/C	V247G	N/A	Damaging	Possibly damaging	
	MLL	11	117860802	G	C/G	E1412Q	N/A	Tolerated	Possibly damaging	
	CAPRIN2	12	30775706	G	A/G	H300Y	N/A	Tolerated	Benign	
	ANG	14	20231996	C	C/T	R145C	N/A	Damaging	Probably damaging	
	ZNF385C	17	37433611	C	C/G	E54Q	N/A	Damaging	Probably damaging	
	SYMPK	19	51037538	C	C/G	K299N	N/A	Damaging	Probably damaging	
101815	L1TD1	1	62445515	#	£­G	--	N/A	Frameshift	N/A	
	S1PR1	1	101477688	C	C/T	A187V	N/A	Tolerated	Benign	
	VAMP5	2	85673721	C	C/G	P94R	N/A	Damaging	Probably damaging	
	YSK4	2	135461930	G	G/T	Q215K	N/A	Damaging	Benign	
	TGFBR2	3	30704879	G	C/G	splice-3	N/A	Damaging	N/A	
	SLC12A7	5	1105496	C	C/G	E1077D	N/A	Not scored	Probably damaging	
	LRRC4C	11	40093714	C	A/C	R235S	N/A	Damaging	Benign	
	GLDN	15	49479862	A	A/G	E301G	N/A	Tolerated	Benign	
	TP53	17	7518988	G	A/G	R196*	N/A	N/A	N/A	
	CD40LG	X	135569109	G	A/G	G198R	rs148594123	Tolerated	Benign	
101919	NASP	1	45845630	#	£­AAG	--	N/A	AA deletion	N/A	
	GRID2	4	94655491	A	A/G	E700G	N/A	Damaging	Probably damaging	
	ADAM29	4	176135362	#	£­A	--	N/A	Frameshift	N/A	
	ABLIM3	5	148543301	G	A/G	R36H	N/A	Damaging	Probably damaging	
	CLK2P	7	23591346	C	A/C	D447Y	N/A	Not scored	Probably damaging	
	LRGUK	7	133474796	C	C/T	A158V	rs187536463	Tolerated	Benign	
	JAK2	9	5012196	#	£­T	--	N/A	Frameshift	N/A	
	TMTC1	12	29560653	#	+T	--	N/A	Frameshift	N/A	
	INHBC	12	56130033	C	C/G	D340E	N/A	Damaging	Probably damaging	
	OAS2	12	111928633	G	A/G	E501K	rs144198504	Tolerated	Benign	
	OR10G3	14	21107916	C	A/C	S267I	rs149233136	Tolerated	Possibly damaging	
	CES1	16	54412917	C	A/C	S186I	N/A	Damaging	Probably damaging	
	GPR114	16	56166890	G	A/G	R509H	N/A	Tolerated	Benign	
	TP53	17	7519004	#	£­G	--	N/A	Frameshift	N/A	
	GINS1	20	25353885	#	£­TA	--	N/A	Frameshift	N/A	
	RPS6KA6	X	83276467	T	C/T	I209V	N/A	Tolerated	Benign	
	DACH2	X	85954517	G	C/G	E402Q	N/A	Tolerated	Probably damaging	
	DIAPH2	X	96241373	C	A/C	Q758K	N/A	Tolerated	Benign	
103048	PAQR7	1	26062707	C	C/T	E71K	rs149765684	Damaging	Probably damaging	
	CYS1	2	10116520	G	A/G	R130C	N/A	Tolerated	Benign	
	PREB	2	27210036	G	A/G	H65Y	N/A	Damaging	Probably damaging	
	CDH9	5	26941944	T	C/T	Q231R	N/A	Tolerated	Benign	
	CNTNAP2	7	146460433	C	C/T	A416V	rs34456867	Tolerated	Benign	
	TMC1	9	74493490	C	C/G	S21*	N/A	N/A	N/A	
	NUP188	9	130771558	G	A/G	A286T	N/A	Damaging	Probably damaging	
	IRX6	16	53919192	C	C/T	R203C	N/A	Damaging	Probably damaging	
	TP53	17	7518960	T	C/T	Y205C	N/A	Damaging	Probably damaging	
	SSH2	17	24999441	C	C/T	R398H	N/A	Damaging	Probably damaging	
	NFATC2	20	49524011	G	A/G	R541W	N/A	Damaging	Probably damaging	
	EFCAB6	22	42303515	C	C/G	E1139Q	N/A	Tolerated	Benign	
	DMD	X	32627155	G	A/G	Q268*	N/A	N/A	N/A	
	CACNA1F	X	48948567	C	C/T	E1959K	N/A	Damaging	Probably damaging	
	TSPYL2	X	53131634	G	C/G	E445D	N/A	Damaging	Benign	
Note: Genomic positions are numbered according to NCBI build 36/hg18. # in the reference allele column indicates that an indel occurs following the given position, and + and – in the column for genotype in tumor indicate short insertion and deletion, respectively. * in the column AA change stands for a stop codon and -- indicates that information for AA change is not provided for indels. N/A in the column rs ID means that the NSSM or indel is not reported before and a SNP ID is provided otherwise. SIFT and PolyPhen2 provide prediction of possible impact of amino acid substitutions and N/A indicates that no prediction is available. NSSM, non-synonymous somatic mutation; Chr, chromosome; AA, amino acid; SIFT, sorting tolerant from intolerant.
