# Supplementary material for: An Old Story Retold: Loss of G1 Control Defines A Distinct Genomic Subtype of Esophageal Squamous Cell Carcinoma
Source: Genomics Proteomics Bioinformatics. 2015 Sep 16;13(4):258–70. doi: 10.1016/j.gpb.2015.06.003 (PMC4610972; doi:10.1016/j.gpb.2015.06.003)
Supplement: Supplementary Table S4 — TP53 mutations identified in 23/46 samples by Sanger sequencing. [file mmc4.rtf]

Table S4  TP53 mutations identified in 23/46 samples by Sanger sequencing
Sample ID	Exon	Nucleotide change	AA change	Mutation type	PolyPhen-2 prediction	
108073	5	176TGC>CGC	C176R	Missense	Probably damaging	
108960	5	154GGC>GAC	G154D	Missense	Possibly damaging	
108987	7	248CGG>TGG	R248W	Missense	Probably damaging	
109596	2	11GAG>CAG	E11Q	Missense	Probably damaging	
110165	6	195ATC>ACC	I195T	Missense	Probably damaging	
110270	4	90TCC>TTCC	Frameshift	Frameshift 	N/A	
		101AAA>AGA	K101R	Missense	Benign	
110274	4	38CAA>TAA	Q38*	Truncating	N/A	
110390	7	236TAC>TGC	Y236C	Missense	Probably damaging	
110852	5	126TAC>GAC	Y126D	Missense	Probably damaging	
110892	4	91TGG>TGA	W91*	Truncating	N/A	
111603	8	266GGA>ACA	G266T	Missense	Probably damaging	
111726	5	175CGC>CAC	R175H	Missense	Possibly damaging	
111820	5	154GGC>GTC 	G154V 	Missense; 	Probably damaging; 	
		176TGC>TTC	C176F	Missense	Probably damaging	
111822	8	282CGG>TGG	R282W	Missense	Probably damaging	
111930	8	286GAA>TAA	E286*	Truncating	N/A	
111944	7	248CGG>CAG	R248Q	Missense	Probably damaging	
111954	4	91TGG>TAG	Q38*	Truncating	N/A	
111958	5	176TGC>TTC	C176F	Missense	Probably damaging	
	8	285GAG>AAG	E285K	Missense	Probably damaging	
112077	5	132AAG>AGG	K132R	Missense	Probably damaging	
112282	7	242TGC>TTC	C242F	Missense	Probably damaging	
112552	5	164AAG>TAG	K164*	Truncating	N/A	
112648	7	253ACC>AACC	Frameshift	Frameshift	N/A	
112887	8	274GTT>GAT	V274D	Missense	Probably damaging	
Note: The nucleotides altered in relative to the reference genome NCBI build 36 are highlighted in bold. AA, amino acid.
