# Supplementary material for: An Old Story Retold: Loss of G1 Control Defines A Distinct Genomic Subtype of Esophageal Squamous Cell Carcinoma
Source: Genomics Proteomics Bioinformatics. 2015 Sep 16;13(4):258–70. doi: 10.1016/j.gpb.2015.06.003 (PMC4610972; doi:10.1016/j.gpb.2015.06.003)
Supplement: Supplementary Table S5 — Top five GO biological processes enriched. [file mmc5.rtf]

Table S5  Top five GO biological processes enriched
GO biological process	P value	Genes 	
Chromosome organization	0.002	TSPYL2, MLL, SMARCD3, NASP, PHF16, TP53, WRN, SYCP2, UBN1, CHD3	
Chromatin modification	0.005	TSPYL2, MLL, SMARCD3, NASP, PHF16, UBN1, CHD3	
Actin filament-based processes	0.013	CAPN10, SORBS1, ANG, DIAPH2, SSH2, MYH7	
Cytoskeleton organization	0.014	KIF2C, CAPN10, SORBS1, ANG, DIAPH2, ABLIM3, DMD, SSH2	
Regulation of neuron apoptosis	0.016	XRCC2, GRID2, TP53, JAK2	
