# Supplementary material for: An Old Story Retold: Loss of G1 Control Defines A Distinct Genomic Subtype of Esophageal Squamous Cell Carcinoma
Source: Genomics Proteomics Bioinformatics. 2015 Sep 16;13(4):258–70. doi: 10.1016/j.gpb.2015.06.003 (PMC4610972; doi:10.1016/j.gpb.2015.06.003)
Supplement: Supplementary Table S6 — Fractions of genomic alterations in 55 ESCCs detected by whole-genome SNP array. [file mmc6.rtf]

Table S6  Fractions of genomic alterations in 55 ESCCs detected by whole-genome SNP array
Patient ID	TP53 mutation	Genome-wide fraction	
		CNG	CNL	CNNLOH	Overall	
99648*	No	0.000	0.015	0.000	0.015	
100036*	No	0.000	0.001	0.000	0.001	
101105*	Yes	0.076	0.085	0.228	0.389	
101506*	Yes	0.001	0.000	0.090	0.091	
101795*	No	0.060	0.037	0.410	0.507	
101815*	Yes	0.106	0.009	0.514	0.629	
101919*	Yes	0.184	0.167	0.406	0.757	
102995*	No	0.002	0.000	0.000	0.002	
103048*	Yes	0.148	0.281	0.494	0.922	
107859	No	0.011	0.001	0.146	0.158	
108073	Yes	0.127	0.093	0.622	0.842	
108607	No	0.080	0.006	0.001	0.087	
108736	No	0.001	0.031	0.000	0.032	
108932	No	0.058	0.024	0.266	0.348	
108960	Yes	0.131	0.344	0.451	0.926	
108987	Yes	0.191	0.200	0.512	0.902	
109121	No	0.191	0.125	0.634	0.950	
109382	No	0.107	0.201	0.000	0.308	
109554	No	0.118	0.038	0.447	0.603	
109596	Yes	0.105	0.066	0.279	0.450	
110165	Yes	0.274	0.290	0.298	0.863	
110197	No	0.068	0.015	0.087	0.169	
110269	No	0.158	0.132	0.501	0.792	
110270	Yes	0.087	0.016	0.469	0.572	
110274	Yes	0.261	0.215	0.444	0.921	
110390	Yes	0.034	0.031	0.393	0.459	
110440	No	0.000	0.008	0.004	0.012	
110850	No	0.000	0.029	0.000	0.030	
110852	Yes	0.193	0.330	0.311	0.834	
110855	No	0.219	0.152	0.343	0.714	
110892	Yes	0.106	0.060	0.654	0.821	
110957	No	0.066	0.041	0.577	0.684	
111531	No	0.216	0.033	0.341	0.590	
111603	Yes	0.193	0.091	0.332	0.616	
111667	No	0.161	0.301	0.238	0.700	
111726	Yes	0.151	0.119	0.287	0.557	
111820	Yes	0.360	0.337	0.270	0.966	
111822	Yes	0.039	0.087	0.449	0.575	
111898	No	0.027	0.012	0.253	0.293	
111926	No	0.054	0.013	0.000	0.067	
111930	Yes	0.077	0.014	0.033	0.124	
111944	Yes	0.157	0.220	0.296	0.673	
111954	Yes	0.098	0.183	0.368	0.648	
111958	Yes	0.185	0.060	0.614	0.859	
111959	No	0.000	0.001	0.001	0.002	
112053	No	0.130	0.076	0.674	0.880	
112077	Yes	0.016	0.009	0.222	0.247	
112282	Yes	0.139	0.190	0.144	0.472	
112325	No	0.042	0.011	0.001	0.054	
112400	No	0.022	0.048	0.002	0.073	
112402	No	0.009	0.028	0.010	0.047	
112552	Yes	0.280	0.380	0.287	0.947	
112644	No	0.006	0.010	0.078	0.094	
112648	Yes	0.078	0.006	0.266	0.351	
112887	Yes	0.079	0.138	0.483	0.700	
Median		0.087	0.048	0.287	0.557	
Note: Genomic alterations in tumor samples were detected using whole-genome SNP array. CNG, copy number gain; CNL, copy number loss; CNNLOH, copy number neutral loss of heterogeneity. * indicates the nine patients with tumor and blood samples subjected to exome sequencing.
