# Supplementary material for: An Old Story Retold: Loss of G1 Control Defines A Distinct Genomic Subtype of Esophageal Squamous Cell Carcinoma
Source: Genomics Proteomics Bioinformatics. 2015 Sep 16;13(4):258–70. doi: 10.1016/j.gpb.2015.06.003 (PMC4610972; doi:10.1016/j.gpb.2015.06.003)
Supplement: Supplementary Table S7 — Fractions of genomic alterations in nine blood samples from ESCC patients detected by whole-genome SNP array. [file mmc7.rtf]

Table S7  Fractions of genomic alterations in nine blood samples from ESCC patients detected by whole-genome SNP array
Sample ID	Genome-wide fraction	
	CNG	CNL	CNNLOH	Overall	
101105B*	0	7.68E−05	0	7.68E−05	
101506B*	0	1.80E−04	0	1.80E−04	
101795B*	0	6.08E−05	0	6.08E−05	
101815B*	1.18E−04	3.33E−04	2.43E−03	0.003	
101919B*	0	0.002	0	0.002	
102995B*	5.73E−05	6.42E−05	0	1.22E−04	
103048B*	0	6.31E−04	0	6.31E−04	
111667B	0	0	0	0	
111820B	0.034	0.019	0.003	0.055	
Note: Genomic alterations in the blood samples of ESCC patients were detected using whole-genome SNP array. CNG, copy number gain; CNL, copy number loss; CNNLOH, copy number neutral loss of heterozygosity. * indicates the blood sample subjected to exome sequencing.
