# Supplementary material for: An Old Story Retold: Loss of G1 Control Defines A Distinct Genomic Subtype of Esophageal Squamous Cell Carcinoma
Source: Genomics Proteomics Bioinformatics. 2015 Sep 16;13(4):258–70. doi: 10.1016/j.gpb.2015.06.003 (PMC4610972; doi:10.1016/j.gpb.2015.06.003)
Supplement: Supplementary Table S8 — Fractions of genomic alterations in blood samples detected using cnvPartition. [file mmc8.rtf]

Table S8  Fractions of genomic alterations in blood samples detected using cnvPartition

Sample ID	Genome-wide fraction	
	CNG	CNL	CNNLOH	Overall	
101105B	2.87E−05	2.75E−05	0.002 	0.002 	
101506B	1.03E−05	4.59E−06	0.002 	0.002 	
101795B	2.64E−05	6.53E−05	0.003 	0.003 	
101815B	1.18E−04	4.08E−04	0.005 	0.005 	
101919B	1.12E−04	8.25E−05	0.003 	0.003 	
102995B	4.59E−06	5.50E−05	0.001 	0.001 	
103048B	1.04E−04	4.59E−06	9.32E−04	0.001 	
111667B	1.79E−04	3.21E−05	0.005 	0.005 	
111820B	0.004 	7.45E−05	0.002 	0.007 	
Note: CNG, copy number gain; CNL, copy number loss; CNNLOH, copy number neutral loss of heterozygosity. 
