# Supplementary material for: An Old Story Retold: Loss of G1 Control Defines A Distinct Genomic Subtype of Esophageal Squamous Cell Carcinoma
Source: Genomics Proteomics Bioinformatics. 2015 Sep 16;13(4):258–70. doi: 10.1016/j.gpb.2015.06.003 (PMC4610972; doi:10.1016/j.gpb.2015.06.003)
Supplement: Supplementary Table S9 — Fractions of genomic alterations on different autosomal arms in 55 ESCC tumor samples. [file mmc9.rtf]

Table S9  Fractions of genomic alterations on different autosomal arms in 55 ESCC tumor samples
Chromosome arm	Fraction on each autosomal arm	
	CNG	CNL	CNNLOH	
1p	0.060	0.042	0.306	
1q	0.087	0.024	0.251	
2p	0.081	0.014	0.252	
2q	0.038	0.067	0.225	
3p	0.099	0.166	0.346	
3q	0.205	0.106	0.196	
4p	0.064	0.124	0.361	
4q	0.078	0.144	0.382	
5p	0.134	0.080	0.265	
5q	0.053	0.125	0.323	
6p	0.064	0.064	0.342	
6q	0.096	0.131	0.226	
7p	0.114	0.070	0.188	
7q	0.133	0.107	0.231	
8p	0.141	0.171	0.217	
8q	0.195	0.149	0.243	
9p	0.155	0.179	0.372	
9q	0.093	0.083	0.461	
10p	0.072	0.082	0.208	
10q	0.064	0.086	0.217	
11p	0.091	0.124	0.235	
11q	0.148	0.176	0.262	
12p	0.132	0.060	0.272	
12q	0.067	0.040	0.313	
13q	0.108	0.121	0.341	
14q	0.175	0.116	0.184	
15q	0.063	0.054	0.256	
16p	0.080	0.056	0.224	
16q	0.072	0.084	0.302	
17p	0.088	0.050	0.493	
17q	0.078	0.025	0.374	
18p	0.124	0.080	0.232	
18q	0.074	0.172	0.271	
19p	0.116	0.053	0.243	
19q	0.122	0.073	0.220	
20p	0.185	0.073	0.182	
20q	0.164	0.060	0.218	
21q	0.106	0.165	0.181	
22q	0.051	0.015	0.182	
Note: CNG, copy number gain; CNL, copy number loss; CNNLOH, copy number neutral loss of heterozygosity. Fractions of gain, loss, and CNNLOH were estimated by dividing the number of SNPs undergoing a specific alteration by the total number of SNPs in the respective chromosome arm. 
